# High-Resolution Imaging Analysis of CT Severity Index in COVID-19 Patients: Impact of Age and Sex

**DOI:** 10.2174/0115734056369445251205082519

**Published:** 2026-04-30

**Authors:** Mahesh Chandra Bhatt, Arun Upmanyu, Subhash Chand Bansal, Ayush Dogra

**Affiliations:** 1 Chitkara, School of Health Sciences, Chitkara University, Rajpura- 140401 Punjab, India;; 2 Chitkara University Institute of Engineering and Technology, Chitkara University, Rajpura- 140401, Punjab, India;; 3 Department of Radiodiagnosis, Chitkara University, PGIMER Chandigarh,160012, Chandigarh, India

**Keywords:** COVID-19, High resolution computed tomography chest, Ground–glass opacity, CT severity score, Severe acute respiratory syndrome coronavirus 2, Real-time reverse transcriptase polymerase chain reaction

## Abstract

**Introduction:**

COVID-19 is a contagious disease caused by severe acute respiratory syndrome coronavirus 2 (SARS-CoV-2). HRCT chest imaging has been widely used during the COVID-19 pandemic. The CT severity score is helpful in assessing disease severity, which may accelerate the diagnostic workflow in COVID-19 patients. The aim of this study is to correlate the chest CT severity score of pulmonary pneumonia in COVID-19 patients with different age groups and sexes.

**Methods:**

This retrospective study investigated the thoracic high-resolution computed tomography (HRCT) findings of 229 laboratory-confirmed COVID-19 patients. The cohort comprised 168 (73.4%) males and 61 (26.6%) females, with ages ranging from 18 to 88 years (median age 47 years). Patients were stratified into four age groups: 18–30, 31–45, 46–60, and >60 years. All patients underwent HRCT of the chest using a Canon Alexion 16-slice CT scanner with a low-dose protocol. Two independent radiologists evaluated the HRCT scans, and a CT severity score was calculated for each patient based on the extent of pulmonary involvement within each lung lobe. Scores were then compared across different age groups.

**Results:**

HRCT chest findings in coronavirus infection included small patchy opacities; ground-glass opacity and consolidation were observed in 83% of patients. The present study indicates a positive correlation between higher CT severity scores and older age groups, as well as male gender, compared with younger and female patients.

**Discussion:**

The study showed that 73.4% of patients were male and 26.6% were female, and that more severe CT lung infection (higher CT severity scores) was significantly associated with male gender. More severe pulmonary infection was also more common in patients above 60 years of age. These findings are in agreement with other studies reporting that COVID-19 infection affects males more severely than females.

**Conclusion:**

HRCT chest imaging provides valuable diagnostic information regarding disease severity, percentage of lung involvement, and extent of disease, which is useful for guiding treatment and prognosis.

## 1 INTRODUCTION

The COVID-19 is a contagious disease caused by severe acute respiratory syndrome coronavirus 2 (SARS-CoV-2) [1]. In December 2019, the first COVID-19 pneumonia cases were identified in Wuhan, China [2] and rapidly transmitted across China and worldwide [2-5]. After nearly four months, on 11 March 2020, this disease was declared a pandemic by the World Health Organization [6]. The coronavirus is transmitted through respiratory droplets, close contact, and *via* the digestive tract [7, 8], leading to acute respiratory distress syndrome and resulting in diffuse alveolar damage [9]. The most common clinical indications of COVID-19 are fever, cough, sore throat, and shortness of breath [10]. In the early days of COVID-19, transmission of infection was controlled by rapid isolation of patients.

The first COVID-19 identification test was introduced by the National Health Commission of the People’s Republic of China (NHCPRC) [11], commonly known as real-time reverse transcription polymerase chain reaction (RT-PCR). To date, it is considered the gold standard for diagnosing COVID-19 infection. Despite its widespread success, the low sensitivity of RT-PCR (71%) [12] and limited testing capacity in the initial stage of the epidemic hindered the quick diagnosis of infected patients. The causes of the low sensitivity of RT-PCR include: (i) premature development of nucleic acid technology; (ii) low patient viral loads; (iii) differences in detection rates from different manufacturers; and (iv) improper patient sampling [12]. Some studies reported that RT-PCR is only 30–60% sensitive [12], and during the early stage of the disease, it is even less sensitive than computed tomography [13].

Chest imaging, including plain chest X-rays and computed tomography, was also considered a confirmatory tool for COVID-19. Chest imaging investigations were commonly performed using chest radiography, computed tomography, and lung ultrasonography [14, 15]. It was observed that in patients with mild symptoms, chest radiographs sometimes produced false-negative results [16]. However, lung ultrasonography has been successfully used to diagnose COVID-19 infection [17-19].

With the advent of high-resolution (HR) computed tomography (CT) chest techniques, better contrast between lung tissues can be achieved. Ground-glass opacities (GGOs) are easily detected by HRCT chest imaging [20]. Chest CT imaging was used as a rapid and early diagnostic tool in Hubei, China, to control COVID-19 infections [21, 22]. According to the sixth edition of the official diagnosis and treatment protocol of the National Health Commission of China, HRCT chest imaging is recommended as one of the diagnostic standards for COVID-19 [23]. The Fleischner Society delivered a multinational consensus regarding the role of HRCT chest imaging during the COVID-19 pandemic, stating that imaging is indicated in suspected COVID-19 patients who have moderate to severe clinical symptoms or present with shortness of breath [24].

In the present work, the CT severity index for 229 COVID-19 infected patients, including 168 males and 61 females, was calculated, and its correlation with age and sex is also reported. The purpose of this study is to correlate the CT severity score of pulmonary pneumonia in COVID-19 patients across different ages and sexes.

The significant features observed in chest CT imaging are ground-glass opacities and consolidations present in the bilateral middle and lower lobes of the lungs [25-28], with 76–85% showing peripheral distribution [25, 26, 29]. Li X *et al*. compared the HRCT chest findings of coronavirus infection with other types of viral pneumonia. All lesions were evaluated for location, distribution, attenuation, number, size, and lobe involvement. The authors found that the characteristic features of 2019-novel coronavirus pneumonia included peripheral distribution, and pleural effusion was not present [30].

**Fig. (1):**
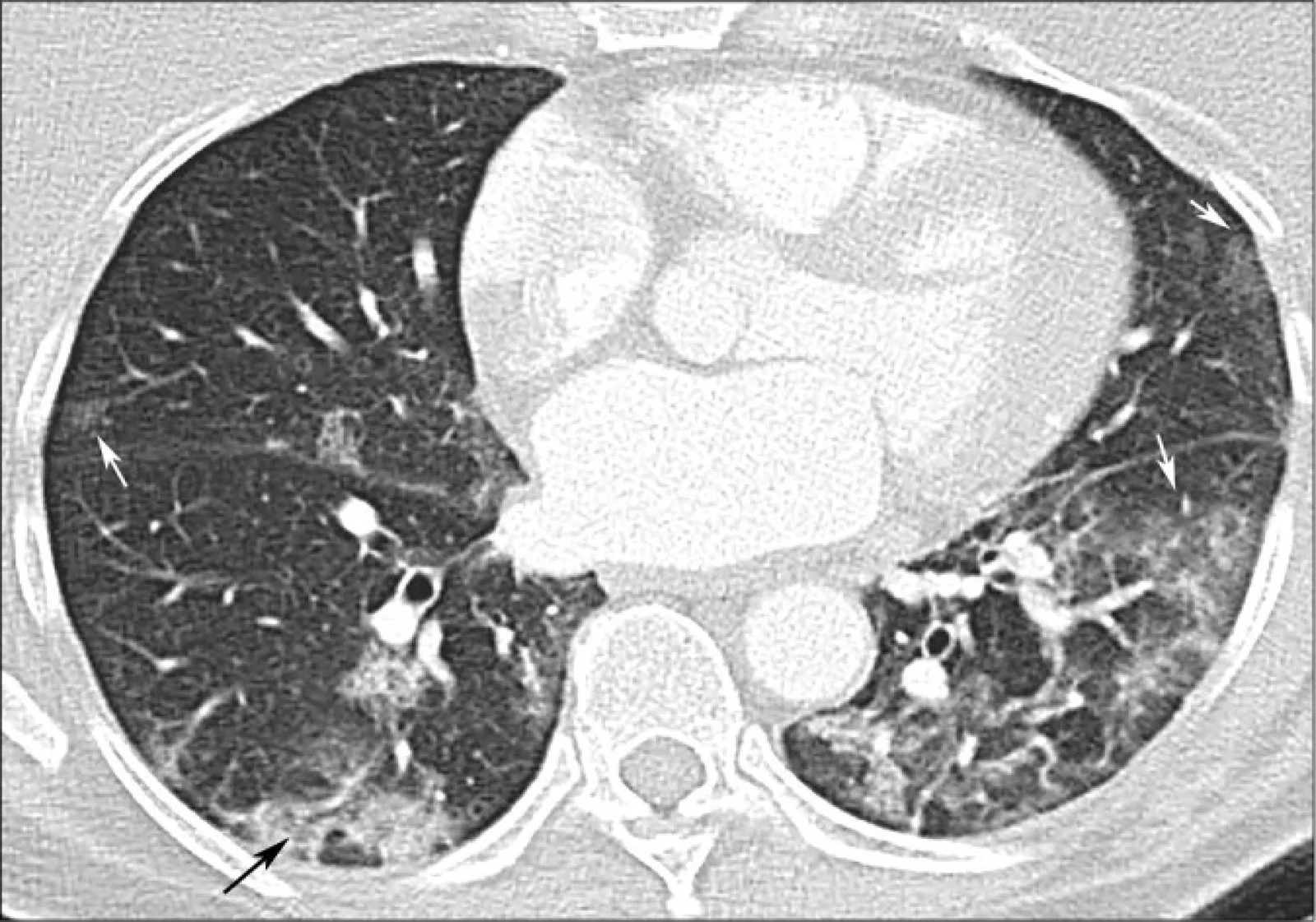
A 61-year-old female with coronavirus disease 2019 pneumonia. Ground glass opacities (white arrows) are predominantly in the left lower lobe, but also in the right middle lobe and the lingula. Right lower lobe consolidation with air bronchogram. Reproduced from [Malguria N, *et al.*, J Clin Imaging Sci, 2021] under the Creative Commons Attribution-Non Commercial- share alike 4.0 License [51].

Bernheim *et al*. retrospectively analyzed the findings of symptomatic infected patients. They found that during the initial days (0–2 days) of infection, patients often had normal chest CT findings; however, as the duration of infection increased, CT findings progressed to ground-glass opacities, consolidation, and greater lung involvement [31]. The second most common feature in chest CT imaging is consolidation (Fig. **1**), which may be present alone or in combination with GGO [25, 29, 32-38].

Dai *et al*. included hospitalized COVID-19 patients in their study. HRCT chest signs and treatment data were collected and analyzed. The majority of patients presented with common symptoms such as fever and cough, along with an epidemiologic history. HRCT chest images showed ground-glass opacities, consolidation, and interlobular septal thickening in bilateral lung fields, with lesions of multiple shapes. HRCT chest findings provided essential information about lung infection [39].

Guan *et al*. retrospectively analyzed follow-up HRCT chest images of COVID-19 pneumonia patients. As the disease progressed, round ground-glass opacities evolved into patchy GGOs, and consolidation increased. When COVID-19 patients recovered completely, air bronchograms and crazy-paving signs markedly decreased [40].

Consolidation indicates more severe disease compared to GGO. Huang *et al*. included 41 COVID-19 patients in their prospective study and showed that, compared to GGO, the presence of consolidation was associated with more severe disease and the need for critical care unit admission [20].

**Fig. (2):**
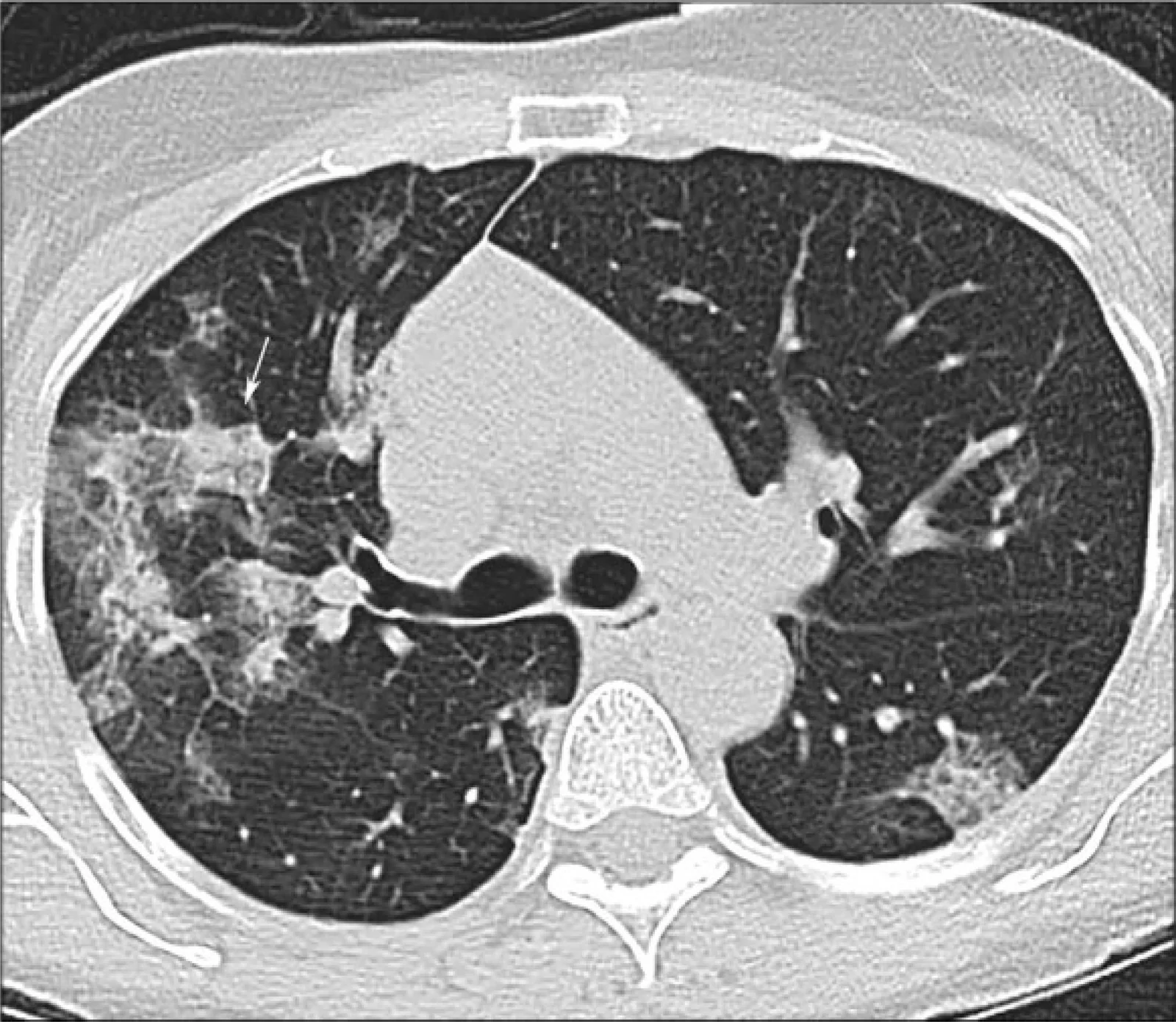
Crazy paving sign seen in the right upper lobe (arrow) of a 58- year-old male. Reproduced from [Malguria N, *et al*., J Clin Imaging Sci, 2021] under the Creative Commons Attribution-Non Commercial- share alike 4.0 License [51].

Crazy paving, including peripheral GGOs with thickened interlobular septa, has been described in chest CT scans of 5–36% of COVID-19 patients (Fig. **2**) [15, 31, 41-43].

The COVID-19 Reporting and Data System (CO-RADS) was used to evaluate the likelihood of pulmonary involvement. In this system, reporting was performed by scoring each of the five lobes based on the distribution of the disease [44]. Francone *et al*. (2020) correlated the CT severity score of total lung involvement with clinical symptoms, disease progression, and laboratory investigations in COVID-19 patients. The CT score showed a strong correlation with laboratory findings, and a higher CT score was associated with increased mortality risk. Thus, the CT score is helpful for assessing disease severity and may assist in accelerating the diagnostic workflow [45].

The percentage of lung infection involvement is scaled in the CT severity index from 0 to 5. The probability of chest infection with respect to the CT severity index is provided in Table **1**. The final score is obtained by adding the scores of each lobe, resulting in a total score out of 25 [45]. The CT severity score is categorized as mild (CTSS 1–7), moderate (CTSS 8–17), and severe (CTSS 18–25).

## 2 AIMS AND OBJECTIVES

The objective of this study was to characterize the typical and atypical chest computed tomography (CT) imaging features of COVID-19 pneumonia. Furthermore, the study aimed to calculate a CT severity score, based on the extent of pulmonary involvement in each lung lobe, and to correlate this score with patient age and sex.

## 3 MATERIALS AND METHODS

### 3.1 Study Design

This is a single-center retrospective study conducted in adult patients diagnosed with COVID-19. All COVID-19 patients above 18 years of age who were admitted to the Government Base Hospital, Almora, Uttarakhand, were included. CTSI was employed to assess the area of lung infection. This study was approved by the Ethical Committee of our hospital (EA/Radiology/21-22). Due to the retrospective nature of the study, patient-informed consent was waived.

### 3.2 Data Collection

Data were collected from the Department of Radiology, Government Base Hospital, Almora (Uttarakhand), from September 2020 to May 2021.

Inclusion criteria: All patients with COVID-19 confirmed positive by either RT-PCR or rapid antigen detection method were included.

Exclusion criteria: Patients who left against medical advice were excluded.

Sample size: The sample size was 229, including 168 males and 61 females (ages 18–88 years).

The Sex and Gender Equity in Research (SAGER) Guidelines were followed.

### 3.3 Imaging Technique

HRCT chest scans of all infected patients were acquired using a Canon Alexion 16-slice CT scanner, following a low-dose CT protocol.

### 3.4 Imaging Interpretation

Experienced radiologists analyzed the chest CT images to determine the CT severity score in each patient. The probability of chest infection with respect to the CT severity index is presented in Table **1**.

### 3.5 Statistical Analysis

Statistical analysis and visualization were performed using the Statistical Package for Social Sciences (SPSS) software, version 21.0 (IBM).

Descriptive statistics were used to summarize the demographic characteristics of the study population, including age and sex. The sample consisted of 229 COVID-19 patients, categorized by age (18–30 years, 31–45 years, 46–60 years, and above 60 years) and sex (male, female). The mean and standard deviation (SD) for age and the frequency distribution for sex were reported. Additionally, the CT severity index (CTSI) was assessed, and its mean and SD were presented.

To examine the impact of age on CTSI, an Analysis of Variance (ANOVA) was performed, comparing CTSI scores across different age groups. Post-hoc comparisons (Tukey’s HSD) were conducted to identify specific differences between groups. The relationship between age and CTSI was further evaluated using Pearson’s correlation coefficient.

Sex differences in CTSI scores were examined using an independent t-test. A *p*-value of < 0.05 was considered statistically significant. The interaction between age and sex was also evaluated to determine any combined effect on CTSI severity. In the present study, the Chi-square test was employed to investigate the relationship between CTSI and sex.

For better understanding, the power analysis of all tests is reported in Table **2**.

The power analysis reported in Table **2** reveals that the power of all the tests is greater than 0.80, indicating that our sample size (229) is adequate (for a medium effect size) for the present study.

## 4 RESULTS

**Fig. (3):**
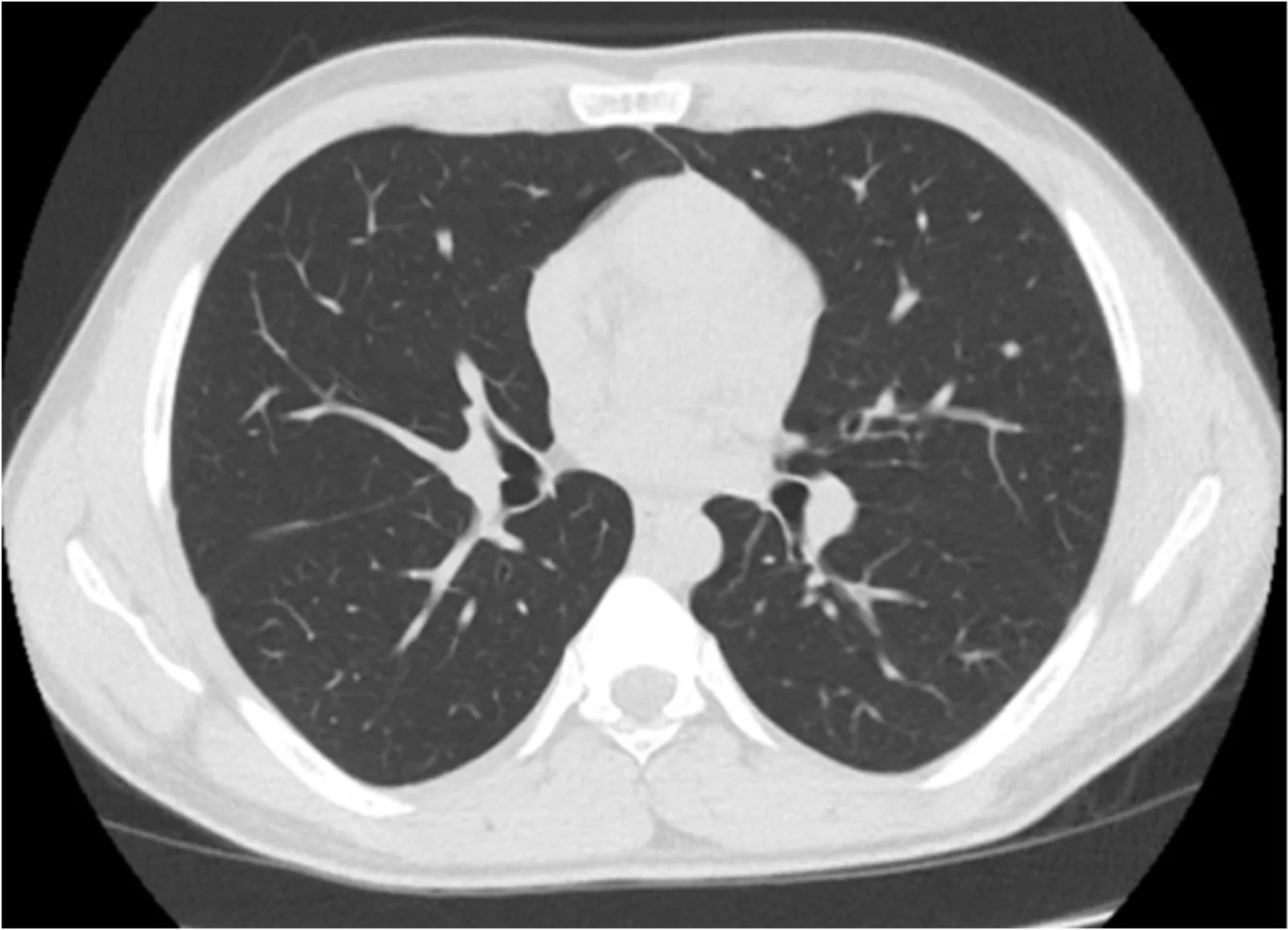
A 21-year-old male patient with a positive RT-PCR test, but no pulmonary infection is seen in the CT images (score 0). (Courtesy: Government Base Hospital, Almora, Uttarakhand).

**Fig. (4):**
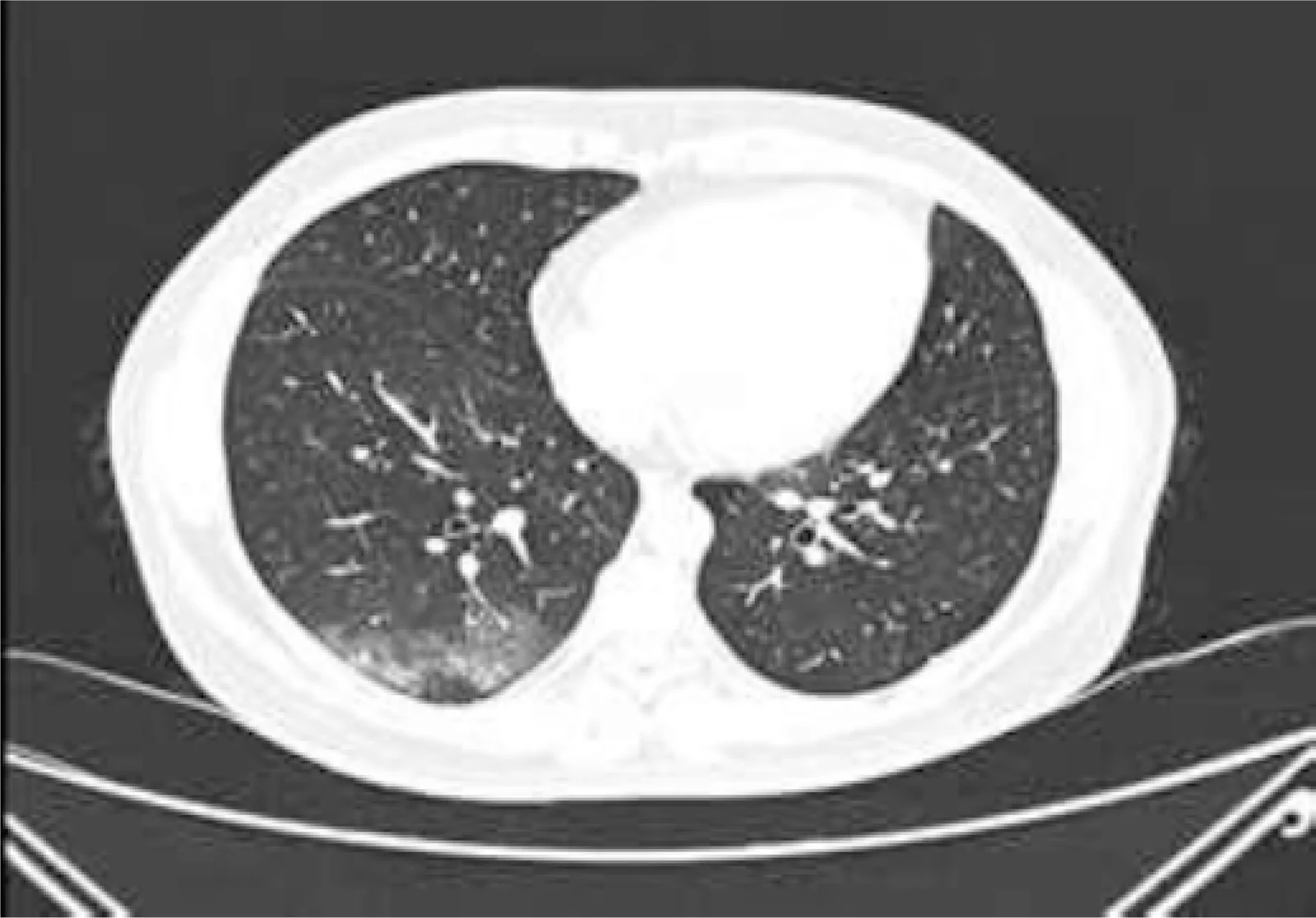
A 32-year-old female patient with COVID-19 pneumonia. Ground-glass opacities are seen in the right upper lobe. Lung involvement is less than 5% (score 1). (Courtesy: Government Base Hospital, Almora, Uttarakhand).

**Fig. (5):**
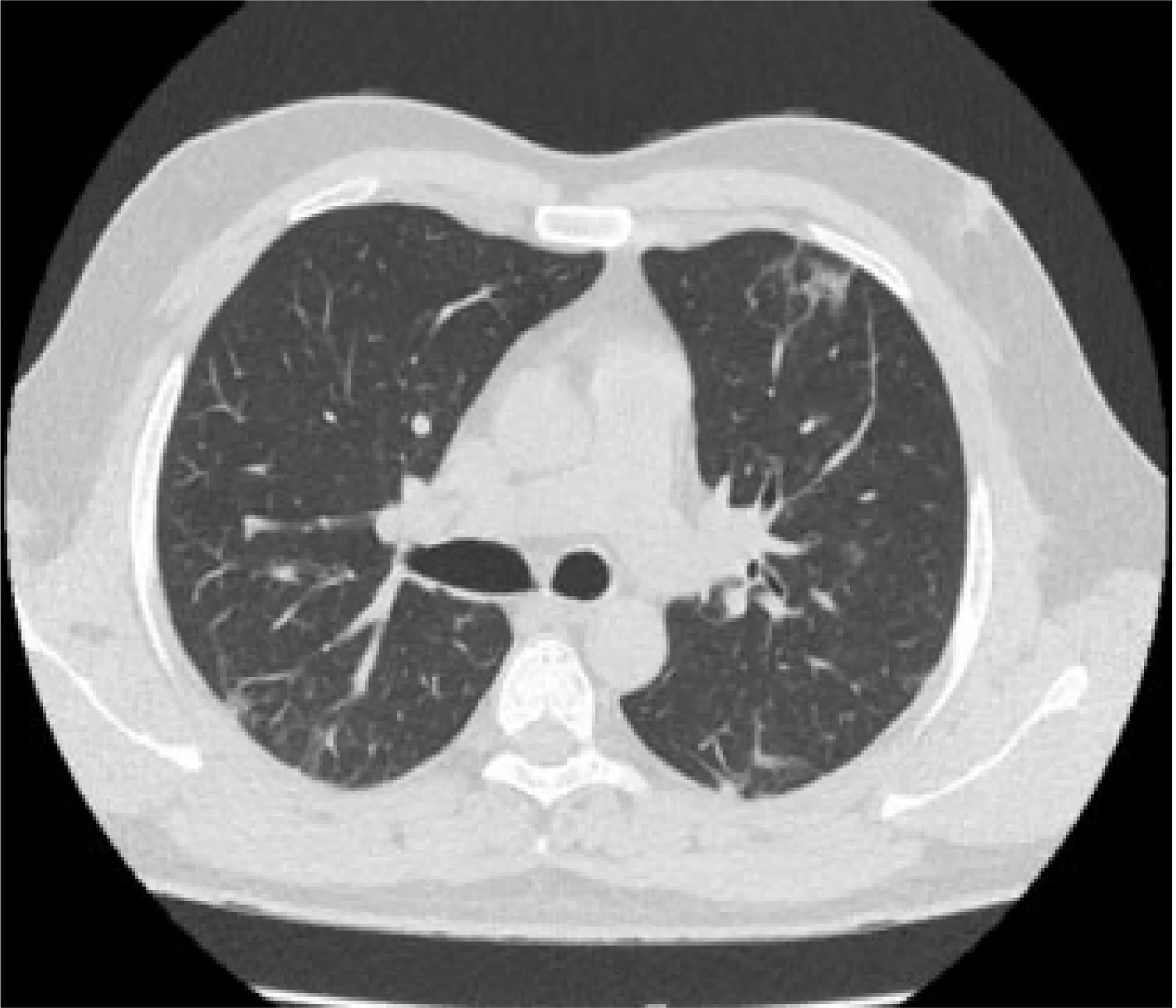
A 40-year-old male patient with COVID-19 pneumonia. Bilateral asymmetrical ground-glass opacities are seen in the right middle lobe and the left upper and lower lobes. Total lung involvement is 5–25% (score 2). (Courtesy: Government Base Hospital, Almora, Uttarakhand).

This study comprised 229 COVID-19 patients, including 168 males (73.4%) and 61 females (26.6%), with ages ranging from 18 to 88 years (median age: 47 years). According to age, patients were categorized into four groups: 18–30 years, 31–45 years, 46–60 years, and above 60 years (Table **3**). Ground-glass opacities were the most common findings in HRCT chest images and were observed in 86.5% of patients, while no pulmonary involvement was seen in 31 patients (score 0, Fig. **3**). Less than 5% lung infection (score 1, Fig. **4**) was seen in 6 patients. Fourteen out of 229 patients had a total lung involvement of 5–25% (score 2, Fig. **5**).

**Fig. (6):**
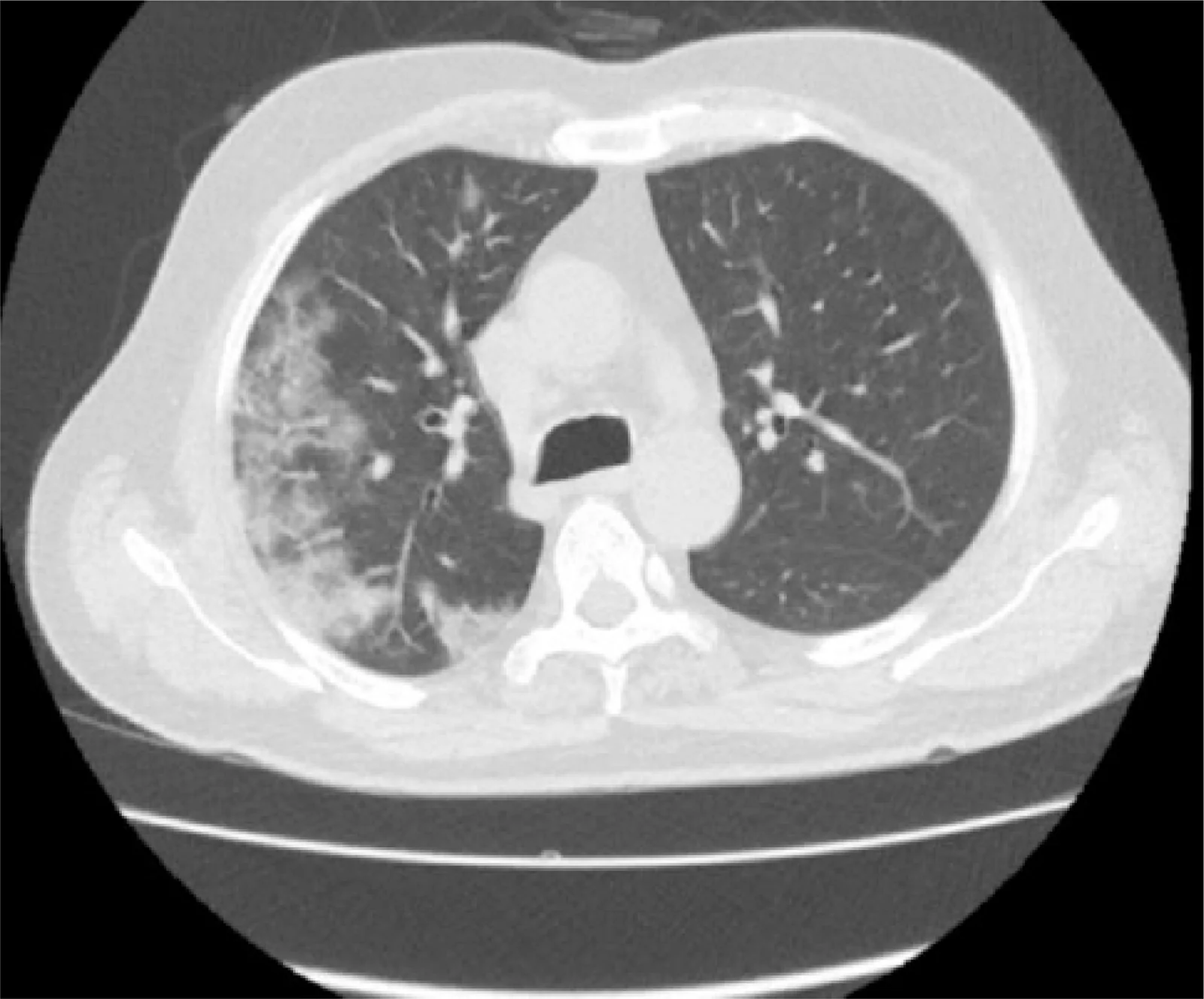
A 46-year-old female patient with COVID-19 pneumonia. Ground-glass opacities are seen in the right upper and middle lobes. Total lung involvement is 26–50% (score 3). (Courtesy: Government Base Hospital, Almora, Uttarakhand).

Fifty out of the total 229 patients had 26–50% lung involvement (score 3, Fig. **6**).

### 4.1 Correlation of CT Severity Score with Age and Sex

**Fig. (7):**
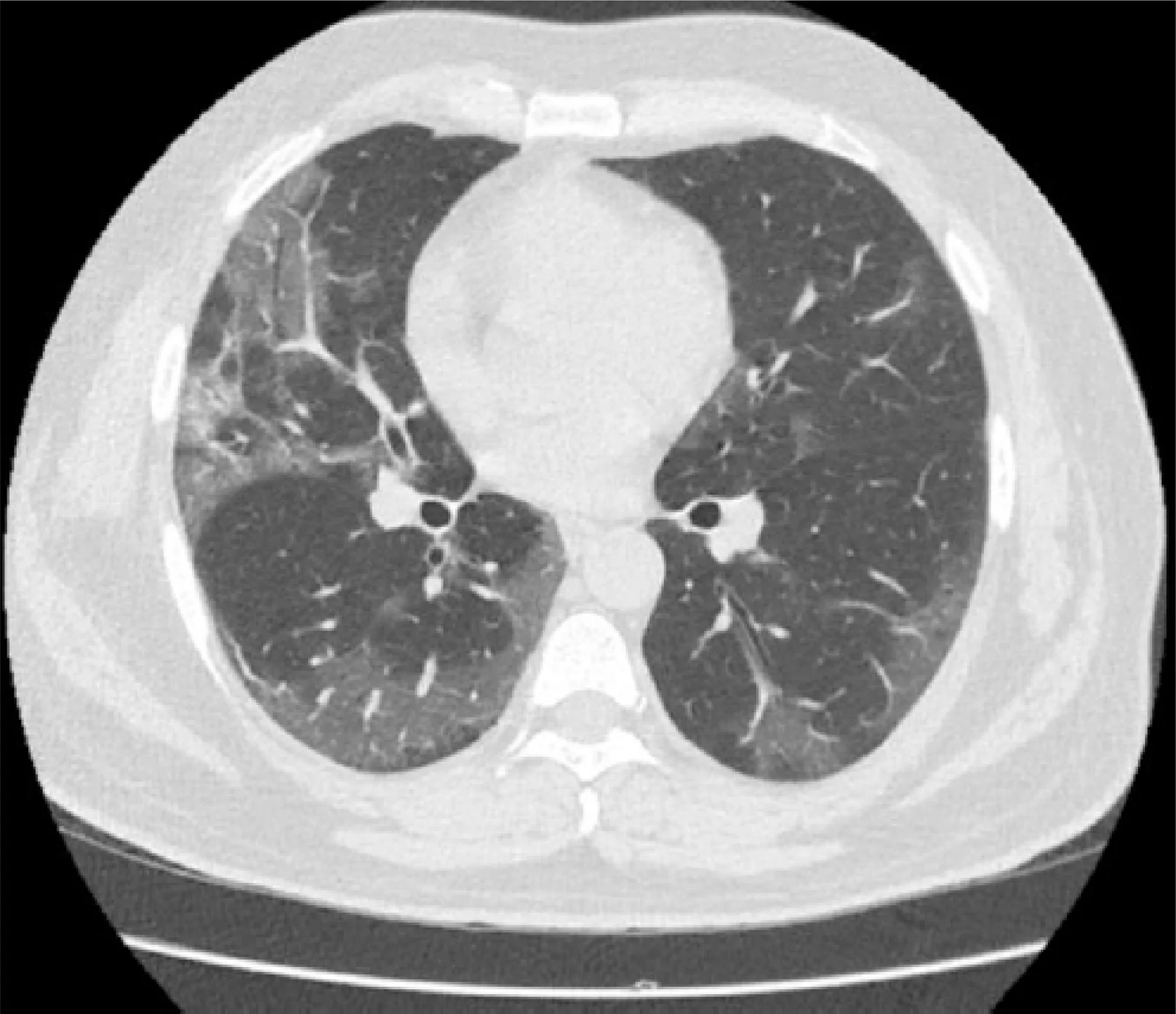
A 50-year-old male patient with COVID-19 pneumonia. Bilateral asymmetrical discrete and confluent patchy areas of GGOs with septal thickening are seen in the bilateral upper lobes, right middle lobe, and bilateral lower lobes, predominantly in the peripheral zone. Lobar involvement is 51–75% (score 4). (Courtesy: Government Base Hospital, Almora, Uttarakhand).

**Fig. (8):**
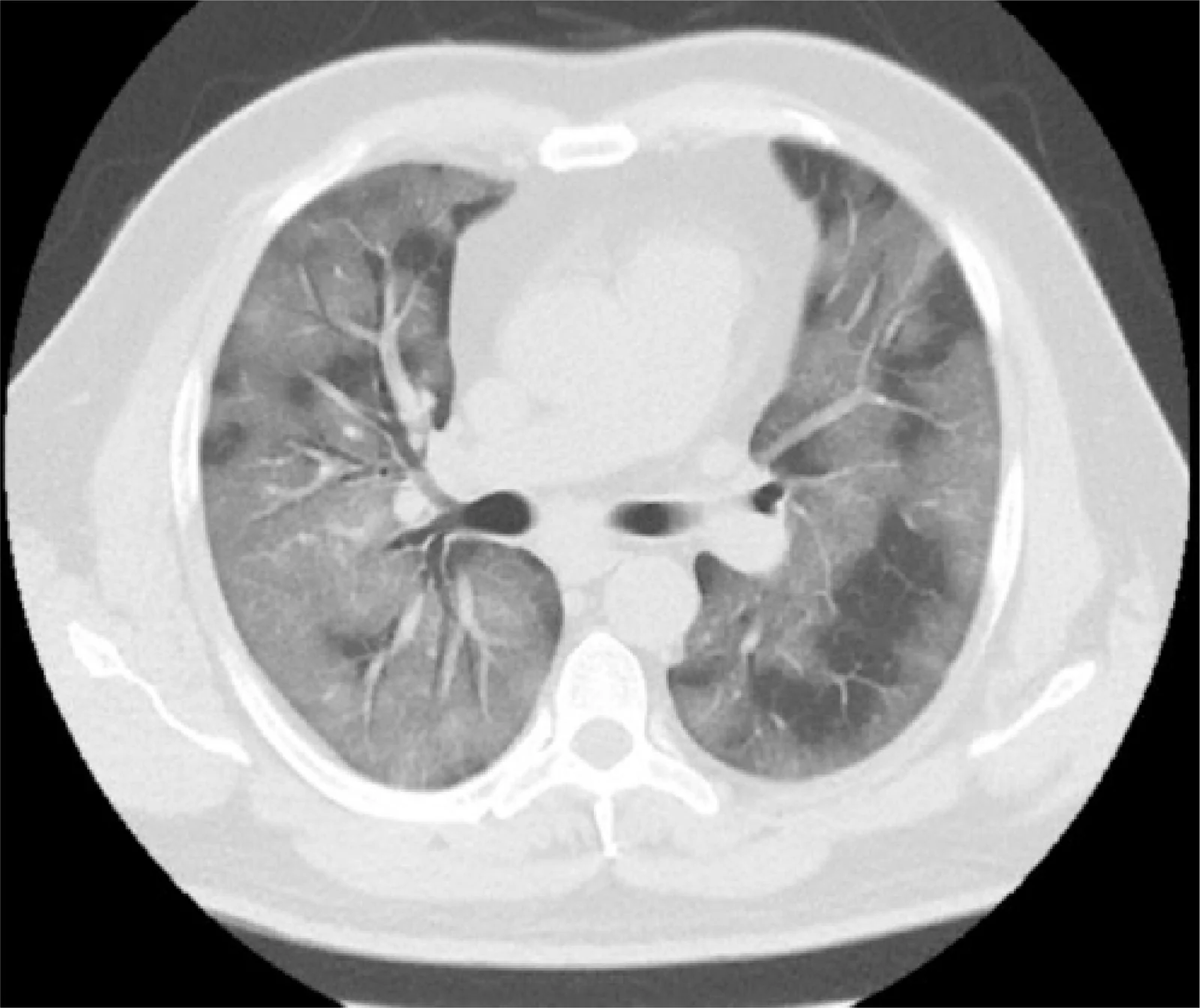
A 60-year-old male patient with COVID-19 pneumonia. Multifocal ground-glass opacities are seen in the bilateral lung parenchyma. Lung involvement is about 90% (CT severity score 5). (Courtesy: Government Base Hospital, Almora, Uttarakhand).

The present study showed a strong positive correlation between CT severity score and male sex (*p*-value = 0.001). Fifty-eight out of 88 patients with a score of 4 (Fig. ****7****) and thirty-seven out of 40 patients with a score of 5 (Fig. **8**) were male. The results are summarized in Table **4**.

There was also a significant correlation between CT severity score and increasing age. Thirty out of 88 patients who had more than 50% pulmonary infection, and 17 out of 40 patients who had more than 75% pulmonary infection, were above 60 years of age (Table **5**).

## 5 DISCUSSION

The present study showed that about 73.4% of patients were male and 26.6% were female, and that more severe CT lung infection (higher CT severity score) was significantly associated with the male gender and with chronic comorbidities such as diabetes, hypertension, cardiovascular disease, and a history of oncologic conditions. This is in agreement with many other studies showing that COVID-19 infection affects males more than females [46, 47].

Severe pulmonary infection was more common in patients above 60 years of age (older age group). An increased number of white blood cells, neutrophils, and C-reactive protein suggests that older patients are more likely to have secondary bacterial infections. Since the coronavirus primarily infects the lungs, pulmonary involvement increases the workload on the heart. Simultaneously, it can raise blood glucose levels, making infection more difficult to control. This challenge is further amplified because older patients are prone to multi-system organ dysfunction. Therefore, complications affecting other body systems should be carefully prevented. This was consistent with the findings reported by Liu *et al*. [48].

In our study, ground-glass opacities were the most common HRCT chest finding and were observed in 198 patients (86.5%). This is consistent with the findings of Parry *et al*. [49], Omar *et al*. in Egypt [46], and Adnan *et al*. [50].

While this study demonstrates a reasonable correlation between chest CT severity scores for pulmonary involvement in COVID-19 patients and patient age and sex, it is important to acknowledge its limitations. The single-center, retrospective design may limit the generalizability of these findings due to potential biases in patient demographics such as age, disease severity, or pre-existing conditions. Furthermore, variations in HRCT chest imaging protocols across different institutions could influence the results and restrict external validity.

Retrospective studies rely on existing records and images, which may contain data gaps, missing clinical information, or incomplete imaging, potentially affecting the comprehen-siveness and reliability of the conclusions. Additionally, such studies often face challenges in controlling for confounding variables, including age, sex, comorbidities, and other clinical factors. These confounders can influence both the severity of COVID-19 and the observed HRCT chest findings, making it difficult to isolate the specific effects of the disease itself.

## CONCLUSION

In this study, we utilized high-resolution imaging to analyze the CT severity index (CTSI) in COVID-19 patients, with a particular focus on the impact of age and sex on disease severity. Our findings indicate that both age and sex significantly influence the CTSI, with older patients demonstrating higher scores, suggesting more severe lung involvement. Furthermore, sex-related differences were observed, with male patients typically exhibiting higher CTSI scores than female patients, which may reflect underlying biological or hormonal factors influencing disease progression.

These results highlight the importance of considering demographic factors such as age and sex when assessing the severity of COVID-19 through CT imaging. HRCT chest imaging is a valuable diagnostic tool for quick and early identification of new cases, benefiting both patients and public health surveillance teams. The CT severity score provides essential information about disease severity, the percentage of lung involvement, and the extent of pulmonary damage, aiding in both treatment planning and prognosis.

Future research should focus on understanding the mechanisms behind these differences and exploring how tailored treatment strategies can be developed based on these demographic factors.

## Figures and Tables

**Table 1:** Probability of chest infection with respect to score.

**Score**	**Lung Infection**
0	No infection in any lobe.
1	< 5% chest infection
2	5 to 25% chest infection
3	26 to50% chest infection
4	51 to 75% chest infection
5	> 75% chest infection

**Table 2:** Power analysis of statistical test.

**Test**	**Purpose**	**Power**
ANOVA	To Compare CTSI across Age Groups	0.898
Independent t-test	To Compare CTSI between Males and Females	0.915
Pearson’s Correlation	Age *vs*. CTSI relationship	1.0
Chi-Square Test	Association between CTSI and Sex	0.995

**Table 3:** Age and sex distribution of COVID-19 patients.

**Sex**	**No.**	**Percentage%**
Male	168	73.4
Female	61	26.6
**Total**	**229**	**100**
**Age group**	**No.**	**Percentage%**
18-30 years	39	17
31-45 years	64	28
46-60 years	67	29.2
Above 60 years	59	25.8

**Table 4:** Association between CT severity score (index) of COVID-19 patients and sex.

**CT Severity Score (Index)/ Pulmonary Infection (%)**	**No.**	**Male No.**	**Female No.**
Score 0 (0%)	31	21	10
Score 1 (<5%)	6	4	2
Score 2 (5-25%)	14	10	4
Score 3 (26-50%)	50	38	12
Score 4 (51-75%)	88	58	30
Score 5 (>75%)	40	37	3

**Table 5:** Association between CT severity score(index) and age groups of COVID-19 patients.

**CTSS(index)**	**18-30 years** **(n=39)**	**31-45 years** **(n=64)**	**46-60 years** **(n=67)**	**Above 60 years** **(n=59)**
Score 0 (n=31)	17	12	2	0
Score 1 (n=6)	2	3	1	0
Score 2 (n=14)	6	2	5	1
Score 3 (n=50)	6	16	22	6
Score 4 (n=88)	7	23	26	32
Score 5 (n=40)	1	08	11	20

## Data Availability

All data generated or analyzed during this study are included in this published article.

## LIST OF ABBREVIATIONS

SARS-CoV-2: Severe Acute Respiratory Syndrome Coronavirus 2
NHCPRC: National Health Commission of the People’s Republic of China
GGOs: Ground-Glass Opacities
CO-RADS: COVID-19 Reporting and Data System
CT: Computed Tomography
CTSI: CT Severity Index
ANOVA: Analysis of Variance

## STANDARDS OF REPORTING

STROBE guidelines were followed
